# Variation in microplastic characteristics among amphibian larvae: a comparative study across different species and the influence of human activity

**DOI:** 10.1038/s41598-024-61432-5

**Published:** 2024-06-12

**Authors:** Michał Szkudlarek, Bartłomiej Najbar, Łukasz Jankowiak

**Affiliations:** 1 Department of Zoology, Institute of Biological Sciences, University of Zielona Góra, Room 504, Profesora Zygmunta Szafrana 1, 65-516 Zielona Góra, Poland; 2 Doctoral School of Exact and Technical Sciences, University of Zielona Góra, al. Wojska Polskiego 69, 65-762 Zielona Góra, Poland; 3 Department of Ecology and Anthropology, Institute of Biology, University of Szczecin, Wąska 13, 71-412 Szczecin, Poland

**Keywords:** Environmental impact, Wetlands ecology, Herpetology

## Abstract

Microplastic pollution is a significant global environmental issue, and impacts span from individual organisms to the entire ecosystems. This study investigated the properties of microplastics in amphibian larvae, shedding light on their environmental interactions and potential ecological consequences. We examined microplastics extracted from amphibian larvae of 10 taxa, sampled from sites experiencing different levels of human impact. Our findings revealed a predominance of blue microplastics and fibres, each comprising 53% of the total microplastics in amphibian larvae. Microplastic fibres were also notably longer than other morphological types of microplastics. Furthermore, we observed variations in the surface area of microplastics among different amphibian families. An interesting observation from our research is the apparent positive relationship between the size of amphibian larvae and the length of granular and flake-shaped microplastics. Conversely, we observed a negative relationship between the length of these microplastics and human environmental impact. These insights significantly contribute to the understanding of microplastic pollution in freshwater environments, highlighting its complexities beyond marine ecosystems. Our research emphasises the intricate relationships between microplastics and freshwater organisms, underscoring the need for comprehensive strategies to mitigate microplastic pollution.

## Introduction

Microplastic pollution has emerged as a global environmental challenge that impacts diverse ecosystems and species. Microplastics (MPs) are either primary or secondary particles in a size range of 1 μm—5 mm^1^ that are made of synthetic or semisynthetic polymers. These anthropogenic pollutants are known to be present across different compartments of the environment where they undergo changes such as weathering, build-up of organisms and organic matter^2^, crystallinity changes, discolouration or fragmentation^3^. More importantly, environmental microplastics are long-lasting and can be easily transported over great distances by air or water^4^. They can interact with biota in many ways, notably through ingestion^5,6^. Within organisms, these particles are able to leach previously adsorbed or absorbed toxic pollutants from water, a phenomenon termed the "Trojan horse effect"^7^. This study focused on a less explored area of microplastic research—the impact on amphibian larvae.

Aquatic free-living amphibian larvae, some of which are indiscriminate omnivores or filter feeders, may ingest and to some extent eliminate^8^ microplastics of various sizes, shapes, colours and chemical compositions. MPs can enter amphibians through their prey^9^, as organisms such as bivalves, zooplankton, and benthic macroinvertebrates have been found to contain these particles^10^. However, the extent of amphibian-mediated trophic transfer of microplastics has not been determined. Amphibian larvae are relevant for studying MPs because: (i) they are ecologically significant primary consumers in lentic ecosystems; (ii) freshly metamorphosed individuals can mediate the transfer of microplastics from aquatic to terrestrial ecosystems^11^; (iii) amphibians are declining worldwide; therefore, understanding the role of microplastic pollution in this phenomenon is important^12^.

Nevertheless, prior research on microplastic pollution has focused primarily on marine environments^13^. Consequently, there is a scarcity of data pertaining to the characteristics of MPs found in freshwater biota, particularly wild amphibian larvae. The majority of amphibian studies investigating the bioaccumulation and the effects of microplastics have relied on indoor exposure experiments^14^ and have focused on anurans^15^ utilising disparate methodologies^16^. However, several studies have investigated the occurrence of these contaminants in wild amphibians, both larval (e.g^17–19^.) and adult individuals (e.g.,^20,21^). Exploring the properties of MPs bioaccumulated by particular species and their respective size classes and/or developmental stages is crucial to experimental ecotoxicological studies as well as modelling (spatial, temporal and ecological) microplastic dynamics. Importantly, the effects of microplastics on animals are, to some extent, type- (chemical-wise), size- and shape-specific (e.g.,^22,23^). Given the nascent nature of this research area, some existing findings remain preliminary or inconsistent.

Here, we attempt to fill this gap by exploring patterns regarding chemical composition, length, surface area, colour and shape of microplastics extracted from different-sized larvae of 10 amphibian species collected across a gradient of human environmental impact (anthropopressure). In particular, we checked whether the lengths of bioaccumulated MPs were related to a proxy of anthropopressure of respective sampling sites, the family (taxonomic rank) of the larvae, or the larval size. We expected the latter to be positively correlated with the length of the bioaccumulated microplastics. Finally, we explored potential relationships between the diversity of MP polymers and larval species.

## Methods

### Sampling, extraction and quality control/assurance

Sampling and extraction methods were detailed in our previous paper^24^. Namely, dead amphibian larvae of various sizes (total *N* = 914 + 20 individuals of unknown origin), representing 10 distinct taxa (9 species + 1 species complex), were opportunistically collected from 23 sites in western Poland, Europe. These sites were locations where mass mortality due to desiccation was observed, including drawdown zones. For more information about the sampling sites see Map 1, Supplementary Fig. S1 online, Supplementary Table S1 online in the previous publication of ours^24^. We obtained the geographic coordinates of each sampling site to conduct spatial analysis, specifically for anthropopressure quantification (see subchapter *Quantification of anthropopressure*). In compliance with ethical standards, we received a permit for specimen collection from the Regional Directorate for Environmental Protection (Permit No. WPN-I.6401.161.2021.JK).

Species identification was conducted with the use of a stereomicroscope and on the basis of morphological criteria given by diagnostic keys^25^.

Amphibian larvae were individually weighed and their total lengths were measured. For microplastic extraction, we treated each larva separately to maintain specificity in the obtained data. Each individual was rinsed with distilled water to eliminate potential microplastics from atmospheric deposition, then cut into pieces and flooded with analytically pure 30% H_2_O_2_ (30 mL) in a 100 mL glass beaker. This process was conducted on an individual basis without pooling. For the digestion of biological matter, glass beakers were incubated for 24 h at 50 °C. The remaining liquid content of each glass beaker, corresponding to a single amphibian larva, was subsequently filtered through a glass fibre filter with a porosity of 1.2 μm (Whatman 1822–047). The purity and quality of each filter were individually verified prior to the procedures. To handle the filters and amphibian larvae, steel tweezers were used.

To prevent sample contamination, the following precautions were taken: wearing a pure cotton lab coat and disposable nitrile gloves, tying back hair, and ensuring that all the equipment and containers were nonplastic and thoroughly prerinsed with distilled water. Laboratories, with minimal personnel and devoid of carpets, were cleaned before each procedure. Furthermore, the processing time of the samples was minimised to reduce exposure to potential contaminants, and the samples were consistently covered if not processed or analysed. A dedicated negative control for tracking background contamination was not employed. However, we anticipate that the impact of background contamination was negligible, as a significant number of samples contained no microplastics (see Supplementary Fig. S3 online in^24^).

### Shape, colour, length and surface area

The methodology of the measurements of particles was described in our previous paper^24^. That is, each filter was placed in a lidded glass Petri dish and inspected under a Nikon ECLIPSE Ni-U microscope (at a magnification of 40 ×), which was connected to a Nikon DS-Fi2 camera (automatic white balance) operating with NIS-Elements Basic Research software. We also used a fibre optic illuminator with the same light intensity for every examination. Particles suspected of being microplastics were identified, counted, and categorised into one of the four distinct morphological types (shapes): fibre (filament), flake (film), fragment, and a granule (sphere). Notably, flakes were distinguished by their markedly unidimensional, flat nature, with edges that are more even and uniform compared to those of fragments. Moreover, each particle was assigned one of the 15 colour categories^26^. The length of each particle’s longest section was measured using the NIS-Elements Basic Research software. For fibres, this measurement captured the entire length, even if coiled. In instances where a fibre bifurcated, the measurement considered the length of the longest strand. The surface areas of flakes, fragments and granules were also manually measured with the aforementioned software. Finally, for record-keeping, photographs of all particles were captured using a Nikon DS-Fi2 camera in tandem with a Nikon ECLIPSE Ni-U microscope (40 × magnification), utilising NIS-Elements Basic Research software.

### Validation and qualitative analysis

**Figure 1:**
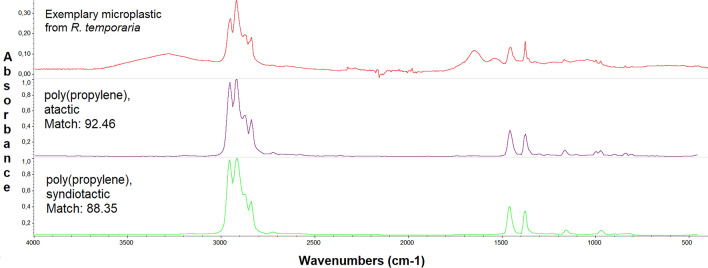
Microplastic spectral analysis. This figure presents the FTIR-ATR spectrum of a microplastic particle extracted from a *Rana temporaria* tadpole. The uppermost graph shows the specific spectral pattern of the sample. Below, the two closest spectral matches from the OMNIC 9.2.106 reference library are displayed. Taken from the OMNIC software, with modifications.

Validation and qualitative analysis of the particles were the same as those described in our previous paper^24^. That is, a representative subset (*N* = 381) of big enough particles from each site-species group, along with negative (samples known not to be made of synthetic polymers) and positive (samples that contain synthetic polymers) controls were analysed using Attenuated Total Reflectance Fourier-Transform Infrared Spectroscopy (ATR-FTIR). This analysis was conducted on a Nicolet™ iS50 FTIR spectrometer, equipped with an ATR module. Particles were individually transferred from the filter surface to the spectrometer's crystal using a steel needle or tweezers. Each measurement involved 50 scans within a wavelength range of 400–4000 cm^−1^ at a resolution of 4 cm^−1^. Between the measurements, the ATR crystal, anvil, and steel instruments were cleaned with 70% ethanol, ensuring complete evaporation of any residual alcohol before subsequent measurements. The acquired spectra were analysed with the OMNIC 9.2.106 software in conjunction with the Open Specy tool^27^. The OMNIC library also contained reference spectra of natural materials. Validation of the samples entailed comparing their spectra against reference spectra of synthetic or semisynthetic polymers available in the libraries (see Fig. 1). A match of at least 60% was needed for the validation. In cases where the match ranged between 60 and 70%, additional manual interpretation of the peaks was undertaken^26^. However, it is worth noting that exposing nylon 6 to a temperature of 50 °C might alter its properties due to a glass transition, potentially resulting in false negatives and an underestimation of this polymer type. Particles that were not analysed but exhibited notable similarities in appearance (such as size, colour and shape) to confirmed microplastic particles were likewise treated as microplastics.

### Quantification of anthropopressure

To quantify anthropopressure, we conducted spatial analyses as detailed in our previous paper^24^. That means, in order to assess the level of human impact (anthropopressure, presumed to be related to microplastic pollution burden) at each sampling location, we utilised a proxy in the form of the share of artificial land cover within a circular buffer with a 1 km radius. The proportion of artificial land cover was calculated using QGIS 3.28.10 'Firenze' software^28^ in conjunction with the CORINE LandCover 2018 dataset^29^.

The choice of artificial land cover as a proxy is grounded in the assumption that areas with higher human activity and infrastructure are more likely to contribute to microplastic pollution. The CORINE LandCover 2018 dataset, provided by the European Environment Agency, offers a comprehensive and accurate representation of land cover across Europe, capturing even minor land use changes^30^.

### Statistical analysis

We analysed microplastics extracted from 934 larvae belonging to the following families and species:

Bufonidae (toads) (*N* = 361):

Common Toad *Bufo bufo* (Bb; *N* = 104 + 20 of unknown origin),

European Green Toad *Bufotes viridis* (Bv; *N* = 120),

Natterjack Toad *Epidalea calamita* (Ec; *N* = 117);

Ranidae (frogs) (*N* = 306):

Moor Frog *Rana arvalis* (Ra; *N* = 88),

Water Frogs *Pelophylax esculentus* complex (Pec; *N* = 90),

Common Frog *Rana temporaria* (Rt; *N* = 128);

Bombinatoridae (*N* = 23):

European Fire-bellied Toad *Bombina bombina* (Bob; *N* = 23);

Salamandridae (newts) (*N* = 244):

Alpine Newt *Ichthyosaura alpestris* (Ia; *N* = 90),

Smooth Newt *Lissotriton vulgaris* (Lv; *N* = 93),

Crested Newt *Triturus cristatus* (Tc; *N* = 61).

Given the significant correlation between the length and weight of amphibian larvae (Pearson correlation coefficient, *P* < 0.001), we used Principal Component Analysis (PCA) to obtain a single variable representing the size of the larvae. This variable demonstrated a strong correlation with both weight and length, showing a correlation coefficient of 0.98 for each. It was thus used as the "size" variable in further analyses.

Mixed-effects normal regression models were used to investigate the effects of family (taxonomic rank, a categorical variable), larval size (a continuous variable from the PCA), shape (a categorical variable), microplastic burden (total number of MPs in an individual), and share of artificial land cover (a continuous variable) on the length and surface area of microplastics. This was performed using the “lme4” package^31^ in R^32^. For these analyses we used 914 amphibian larvae of known origin. The model accounted for variations across sampling sites and species within those sites. By incorporating random effects (syntax: 1 + size | family:site), we accounted for potential variations in both the intercept and size effects across different families and sites. We also introduced interactions into the models: shape × size and shape × share of artificial land cover. Prior to the analyses, the dependent variables (length and surface area) and the independent variable (larval size) underwent logarithmic transformation. Diagnostics confirmed the log length model's compliance with normality by QQ plot (Supplementary Fig. S1 online) and homogeneity assumptions for shape, Levene's test (*F* = 1.067, *p* = 0.362). For the area variable, which violated normality, a Box-Cox transformation (*λ* = − 0.141) was used and produced area_transformed variable which addressed the normality issue (Supplementary Fig. S2 online). Levene's test of this variable for shape also indicated homogeneity (*F* = 0.333, *p* = 0.717). To assess the effects of the terms, we employed a chi-square test to compare the full model with a reduced version that omitted the focal variable. This was achieved using the drop1() function in R, which allowed us to systematically remove each term from the model and compare the resulting models with the original model based on the Akaike Information Criterion (AIC). Nonsignificant interactions were not adopted in the models.

We presented the estimated coefficients, standard errors, and p-values to elucidate the relationships between the independent and dependent variables. Additionally, we investigated potential differences between the families by utilising post-hoc Tukey tests.

For the frequency analysis of (i) the shape of the microplastics for each species, (ii) the colour of each shape of the microplastics, (iii) the colour of the microplastics for each species, and (iv) the particles' chemical composition for each species, we carried out a nonparametric analysis using ANOSIM, a multilevel pattern analysis tool available in the *vegan* package^33^. In this analysis, data derived from the examination of all 934 amphibian larvae, including those of unknown origin, were utilized. ANOSIM is a nonparametric ANOVA-like test that assesses dissimilarities between groups^34^. It computes R values that indicate the level of similarity. R-ANOSIM values greater than 0.75 indicate distinct groups. Values between 0.50 and 0.75 suggest that groups are separate but have some overlap. Values between 0.25 and 0.50 indicate that groups are distinct but with significant overlap, while values lower than 0.25 denote minimal dissimilarities between the groups^35^. To identify differences in shape, colour, and chemical composition, we performed a multipattern analysis, specifically, the Indicator Species Analysis^36^, available in the *indicspecies* package^37^. This method finds similar groups of given categorical variables. For this analysis, we used 99 permutations and data from 934 specimens.

## Results

As previously reported, microplastic pollution was detected at every sampling site and in each taxon^24^, demonstrating the widespread nature of this phenomenon. For data on particle count per each species see Supplementary Table S1 online. For the details on interspecific differences in microplastic pollution and the diversity of morphological types (shapes) of the particles in relation to a proxy of anthropopressure, refer to our previous paper^24^.

### Shape

**Figure 2:**
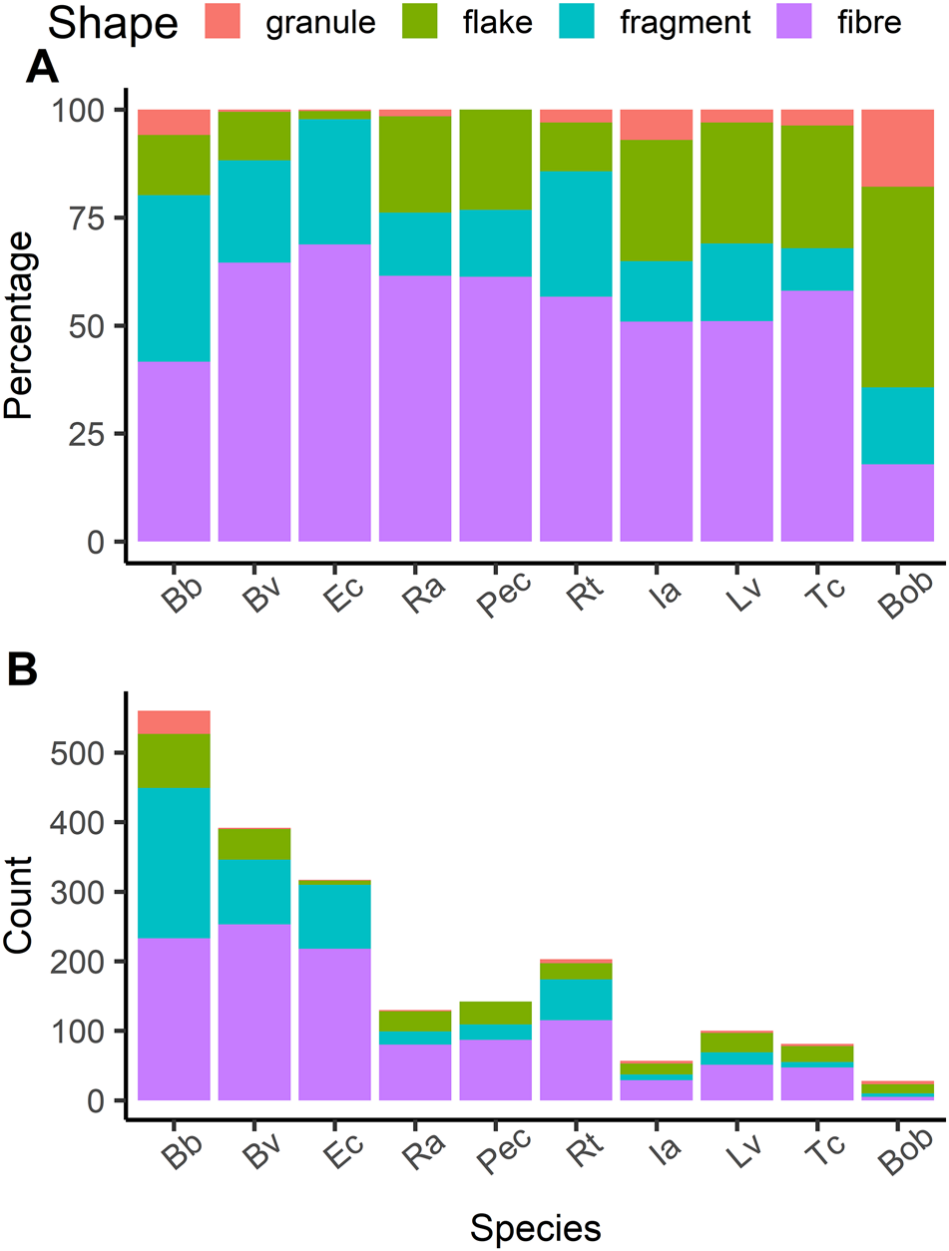
Distribution and abundance of microplastic shapes across amphibian species of all 934 individuals. Panel A illustrates the shares of different microplastic shapes found within each respective species. Panel B shows the abundance of each shape per species. The species abbreviations used are as follows: Bb—Common Toad (*Bufo bufo*); Bv—European Green Toad (*Bufotes viridis*); Ec—Natterjack Toad (*Epidalea calamita*); Ra—Moor Frog (*Rana arvalis*); Pec—Water Frogs (*Pelophylax esculentus* complex); Rt—Common Frog (*Rana temporaria*); Ia—Alpine Newt (*Ichthyosaura alpestris*); Lv—Smooth Newt (*Lissotriton vulgaris*); Tc—Crested Newt (*Triturus cristatus*); Bob—European Fire-bellied Toad (*Bombina bombina*). This artwork was created with the ggplot2 package visualisation tool in R.

Among the four distinct morphological types (shapes) of microplastics, fibres were the most prevalent, while granules were the least common (averaging about 53% and 4%, respectively; see Fig. 2).

The ANOSIM analysis, designed to evaluate dissimilarities, revealed a significant effect of species on the distribution of MP shapes (ANOSIM statistic R: 0.1079, *p* = 0.001). Nonetheless, the observed dissimilarity among species indicated only marginal separation. Furthermore, multilevel pattern analysis identified four groups of species wherein certain microplastic shape/s were more prevalent than in other taxa (for all comparisons, *p* < 0.05): (1) Bb: fragment; (2) Bb + Bob: granule; (3) Bb + Bv + Ec: fibre; (4) Bb + Bv + Pec + Ra + Ia + Lv + Tc + Bob: flake.

### Length

Lengths in the longest section of the plastics extracted from amphibian larvae ranged from 12 to 5700 μm, with 3 fibres exceeding the commonly accepted upper limit for microplastics − 5000 μm^38^. Nonetheless, we chose to include them in the analyses to provide a more complete picture.

The results from the model without interaction showed that only the morphological type (shape) was significant (df = 3, DenDf = 1812.417, *F* value = 908.164, *p* < 0.001; see Supplementary Table S2 online). On average, the fibres were the longest (avg. 840 μm), and post-hoc comparisons revealed significant differences from all the other shapes (flake, granule, fragment; all comparisons *p* < 0.001; see Supplementary Table S3 online). The average granule and flake measured 109 and 95 μm in length, respectively, both longer than the average fragment at 66 μm (*p* < 0.005 for both). However, there was no statistically significant difference in length between a granule and a flake (*p* = 0.814; see Supplementary Table S3 online).

**Figure 3:**
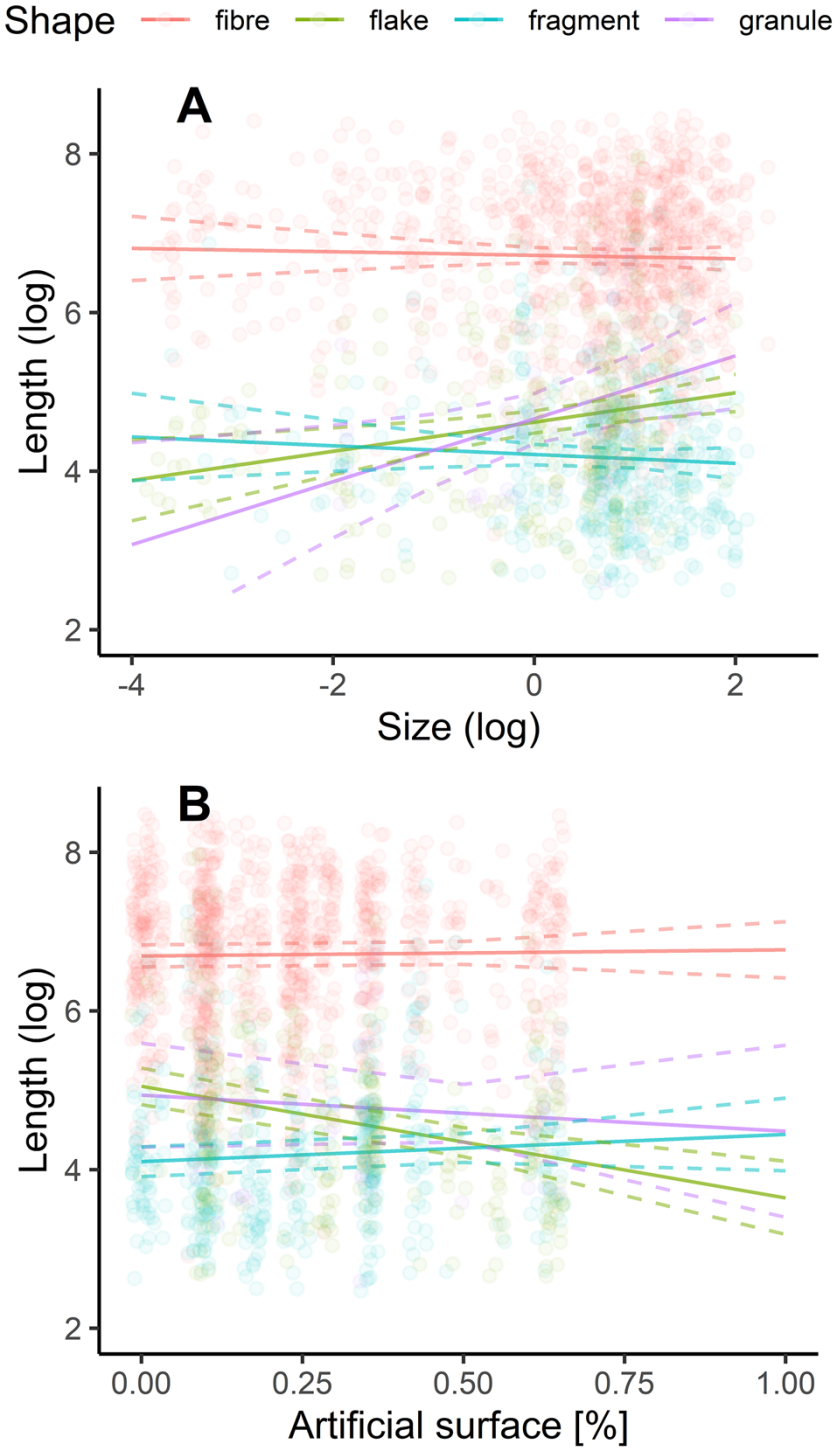
Microplastic length and its relationship with larval size and a proxy of anthropopressure. Panel A displays the relationship between the length of various morphological types of microplastics and the size of amphibian larvae. Panel B explores the connection between microplastic length and a proxy of anthropopressure, again differentiated by the morphological types of the microplastics. Dashed lines represent boundaries of the 95% confidence interval. This artwork was created with the ggplot2 package visualisation tool in R.

The impact of other variables, such as the larva’s family (taxonomic rank), its size, its microplastic burden, and the proportion of artificial land cover within buffers, were not significant (all p > 0.005; see Supplementary Table S2 online). However, the following interactions were significant within the model: (i) shape of MP and size of larva (df = 3, DenDf = 1803.605, *F* value = 8.507, *p* < 0.001), and (ii) shape of MP and proportion of artificial land cover (df = 3, DenDf = 1804.815, *F* value = 8.680, *p* < 0.001) (see Supplementary Table S4 online). Both interactions are depicted in Fig. 3A and B, respectively. Notably, according to this model, larval size had a significant positive effect on the length of a granule and a flake, but not on the length of a fibre or a fragment (see Fig. 3A). The second interaction (see Fig. 3B) indicates that an increase in the proportion of artificial land cover within buffers (a proxy of anthropopressure) reduces the length of a flake and a granule.

### Surface area

Utilising a camera attached to a microscope, in conjunction with NIS-Elements Basic Research software, we measured the surface areas of all the microplastics, excluding fibres, from 2D photographs. This encompassed flakes, granules, and fragments.

On average, the granules possessed the largest surface area (avg. 4033 μm^2^). Post-hoc comparisons (see Supplementary Table S5 online) demonstrated that this value was significantly greater than that of a fragment (avg. 1140 μm^2^; *p* < 0.001). The mean surface area of the flakes was 3025 μm^2^, which was also significantly greater than that of the fragments (*p* < 0.001). However, no statistically significant difference was observed between a flake and a granule (*p* = 0.528).

**Figure 4:**
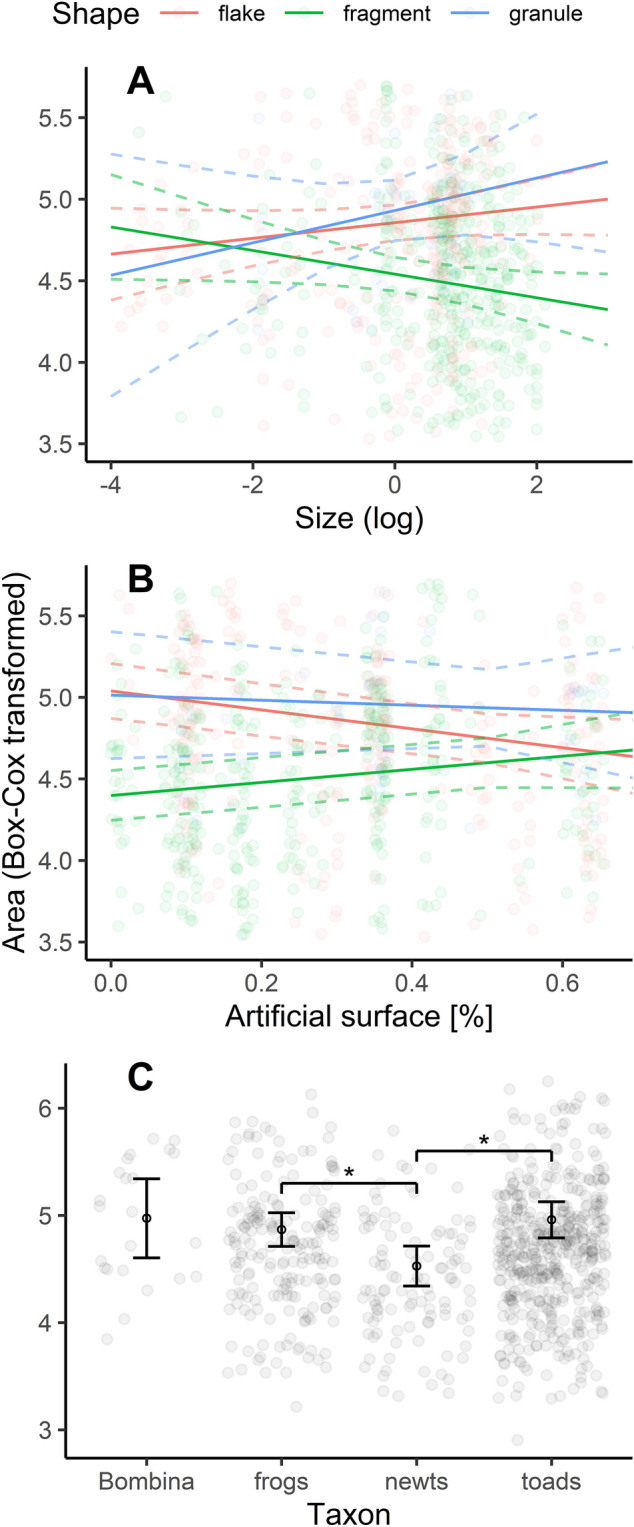
Surface area of microplastics relative to larval size, proxy of anthropopressure and family (taxonomic rank). Panel A illustrates the relationship between the surface area of variously shaped microplastics and the size of amphibian larvae. Panel B shows the relationship between the surface area of microplastics and a proxy of anthropopressure. Panel C presents a boxplot comparison of the surface area of microplastics extracted from larvae of different amphibian families (taxa): Bombinatoridae (European Fire-bellied Toad *Bombina bombina*), Ranidae (frogs), Salamandridae (newts), and Bufonidae (toads). Asterisk denotes *p* < 0.05. Dashed lines represent boundaries of the 95% confidence interval. This artwork was created with the ggplot2 package visualisation tool in R.

The results from testing the model without interactive terms (see Supplementary Table S6 online) indicated that the shape of the microplastic significantly influenced its surface area (df = 2, DenDf = 774.116, *F* value = 33.459, *p* < 0.001). Additionally, we discerned a significant effect of the family (taxonomic rank) within the model (df = 3, DenDf = 21.981, *F* value = 4.470, *p* = 0.014). Post-hoc analyses (see Fig. 4C; Supplementary Table S7 online) revealed that MPs extracted from toad (Bufonidae) tadpoles had a notably larger surface area than those obtained from newt (Salamandridae) larvae (*p* = 0.033). Similarly, MPs isolated from frog tadpoles displayed a significantly larger surface area than those extracted from newt larvae (*p* = 0.045) Other comparisons were not statistically significant (p > 0.05).

Furthermore, we identified two interactions that were statistically significant within the model (see Supplementary Table S8 online): (i) the shape of microplastics and larval size (df = 2, DenDf = 751,866, *F* value = 6.128, *p* = 0.002), and (ii) the shape of MP and the proportion of artificial land cover surrounding sampling sites (df = 2, DenDf = 760.710, *F* value = 9.678, *p* < 0.001). Both interactions are depicted in Fig. 4A,B, respectively. Interestingly, we found that larval size positively influenced the surface area of both the granules and flakes, yet it had a negative effect on the surface area of the fragments. The second interaction showed that an increase in the proportion of artificial land cover within buffers (a proxy of anthropopressure) reduced the surface area of both flakes and granules. In contrast, for fragments, this effect was inverted: their surface area increased.

### Colours

All 15 colour categories were represented in the microplastics analysed in our study. Exemplary specimens of each colour category can be found in Supplementary Figure S3 online.

#### Colours of microplastics based on their shape

With the use of a database of all the microplastics, we analysed the variation in colour composition across their different morphological types (shapes).

**Figure 5:**
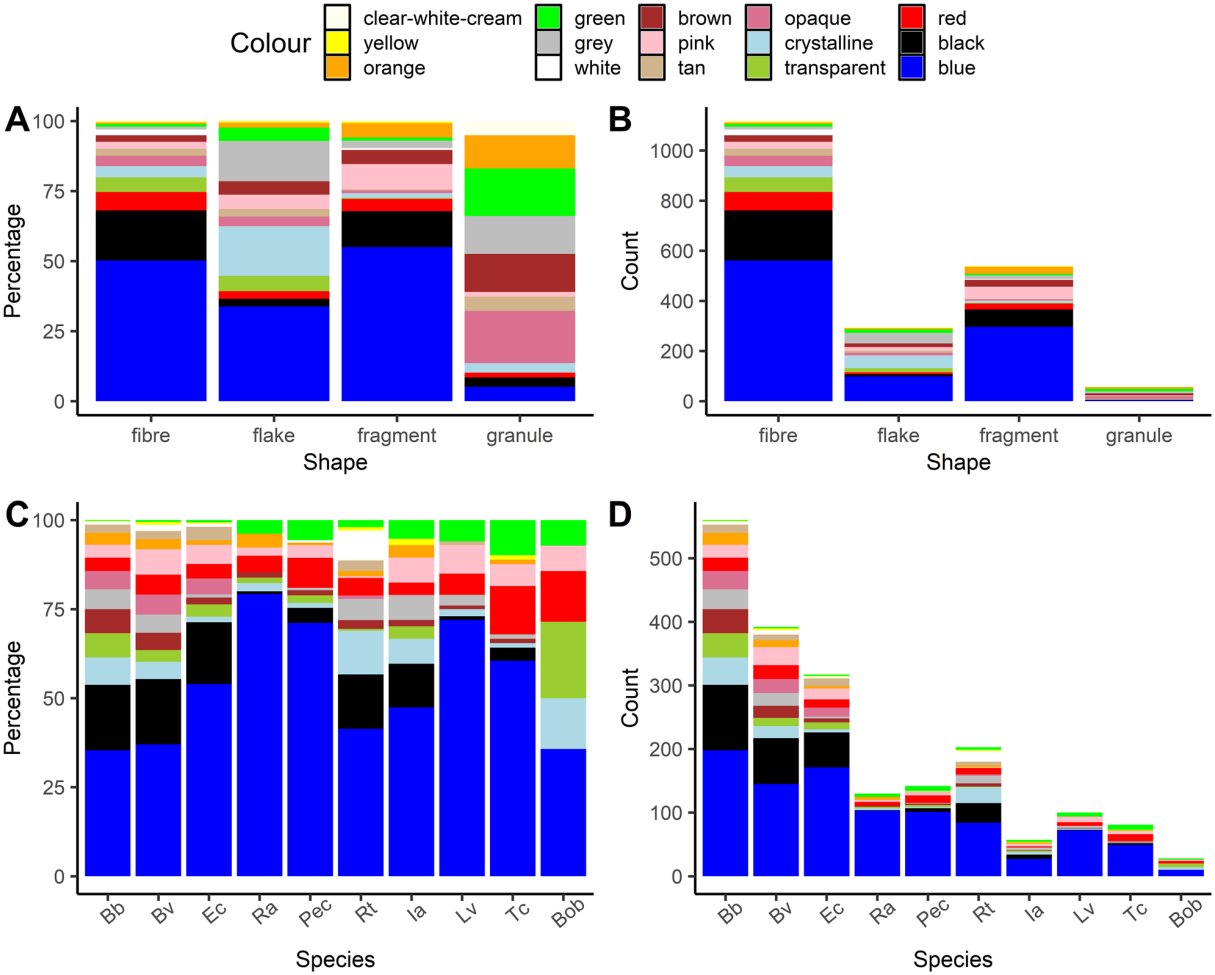
Colour distribution of microplastics in amphibian larvae (*N* = 934). Panel A shows the distribution of colours across different shapes of microplastics. Panel B illustrates the abundance of variously coloured microplastics per their shape. Panel C depicts the proportion of each colour of microplastics in larvae of different amphibian species. Panel D presents the abundance of differently coloured microplastics within each amphibian species. Species abbreviations are consistent with those in Fig. 2. This artwork was created with the ggplot2 package visualisation tool in R.

The ANOSIM (dissimilarities) analysis revealed the significant effect of the shape of microplastics on the colour distribution (ANOSIM statistic R: 0.119, *p* = 0.001). However, the extent of dissimilarity between shapes suggested marginal differentiation. Furthermore, the multilevel pattern analysis identified seven groups of MP shapes wherein specific colour/s were more common (*p* < 0.05) than in other morphological types (see Fig. 5A): 1) fibre: white; 2) flake: crystalline; 3) fragment: pink; 4) granule: opaque, green, clear-white-cream, brown; 5) fibre + flake: transparent; 6) fibre + fragment: blue, black, red; 7) flake + granule: grey. Naturally, with abundances (see Fig. 5B.), these specificities are less pronounced.

#### Colours of microplastics extracted from various taxa

Blue was identified as the most common colour of microplastics across all taxa, with an average representation of 53.4%. Conversely, yellow and clear-white-cream were the least frequent colours, averaging 0.5% and 0.2%, respectively (see Supplementary Table S9 online).

The ANOSIM (dissimilarities) analysis, revealed a nonsignificant effect of larval species on colour distribution (ANOSIM statistic R: 0.008, *p* = 0.175). This suggests a homogeneity in the colour composition of microplastics extracted from different species. Furthermore, the multilevel pattern analysis identified eight groups of species wherein specific microplastic colour/s were observed more frequently (*p* < 0.05) than in other species (see Fig. 5C): (1) Rt: white; (2) Bb + Bv: brown; (3) Bb + Bob: transparent; (4) Bb + Bv + Ec: opaque; (5) Bb + Bv + Ec + Rt: black, tan; (6) Bb + Bv + Rt + Ia: grey; (7) Bb + Bv + Rt + Bob: crystalline; (8) Ec + Pec + Ra + Lv: blue. And again, when looking at the count data (Fig. 5D), these particularities are not clearly visible.

### Chemical composition

A total of 17 synthetic polymers and six other substances were identified. The identification of a synthetic polymer confirmed the microplastic identity, while the detection of other substances denied it. Among the synthetic polymers, the most common were polyethylene (PE, 33%, commonly used as a packaging material), polypropylene (PP, 24%, another common packaging material), and poly(ethylene:propylene:diene) (EPDM, 22%, a type of rubber).

**Figure 6:**
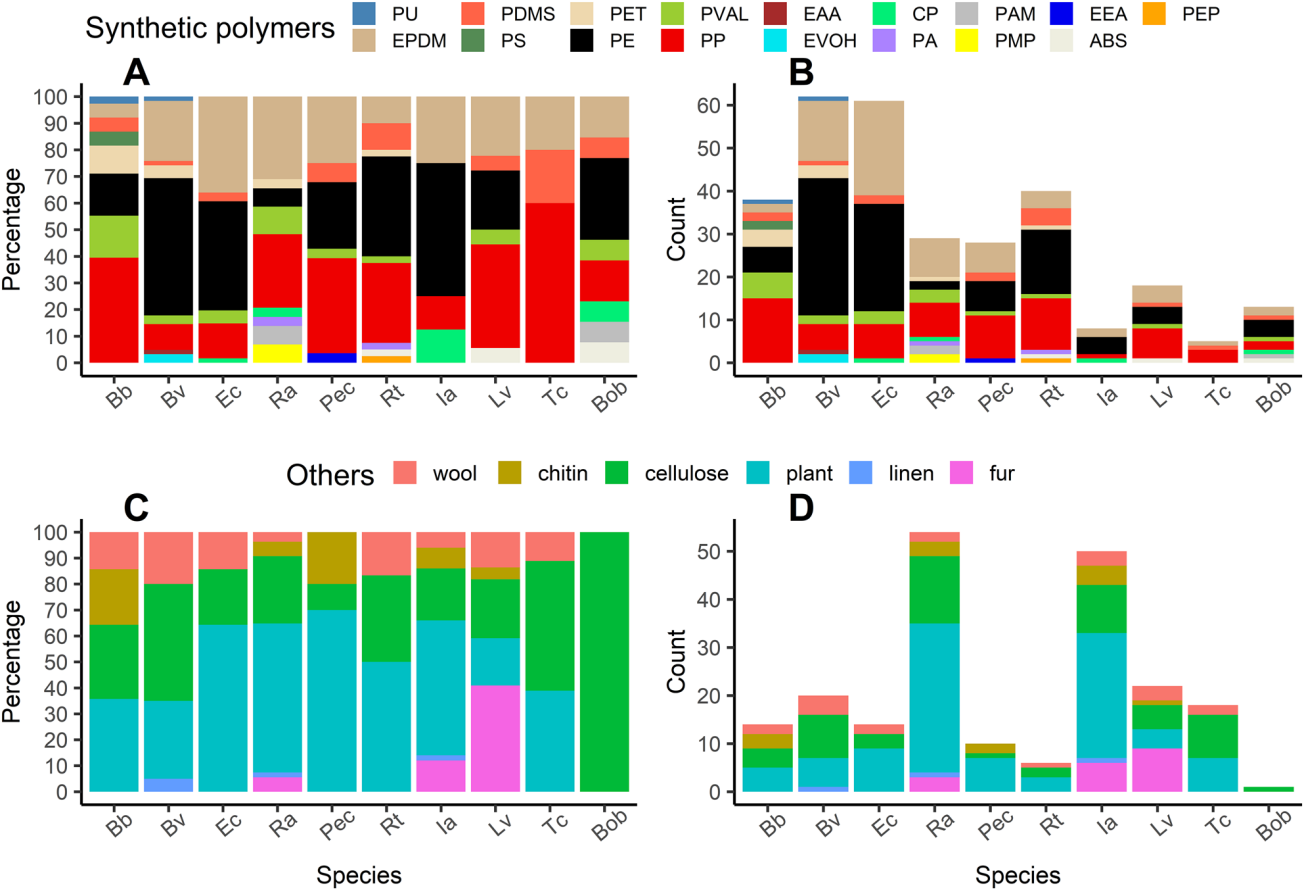
Chemical composition of the studied samples. Shares (**A** and **C**) and total counts (**B** and **D**) of respective subtypes of the two different types of substances: synthetic polymers (**A** and **B**) and other compounds (**C** and **D**) across the studied species. Abbreviations of synthetic polymers: PU—polyurethane; PDMS—polydimethylsiloxane; EPDM—poly(ethylene:propylene:diene); PS—polystyrene; PE—polyethylene; PET—polyethylene terephthalate; PVAL—poly(vinyl alcohol); PP—polypropylene; EAA—ethylene acrylic acid; EVOH—ethylene vinyl alcohol; CP—cellophane; PMP—polymethylpentene; PAM—polyacrylamide; EEA—ethylene ethyl acrylate; ABS—acrylonitrile butadiene styrene; PEP—poly(ethylene:propylene); PA—polyamides (incl. nylon). Species abbreviations are the same as in Fig. 2. This artwork was created with the ggplot2 package visualisation tool in R.

An ANOSIM analysis assessing dissimilarities revealed the significant influence of the species on the distribution of synthetic polymers (ANOSIM statistic R: 0.079, *p* = 0.001), although the interspecific differences were notably subtle. The multilevel pattern analysis indicated one group of species in which a particular synthetic polymer was detected more frequently (*p* < 0.05) than in other species (see Fig. 6A): 1) Bv + Ec + Rt + Ia + Bob: PE.

The data in Fig. 6B, which enumerates the detections of specific synthetic polymers across different species, underscores the variances in synthetic polymer presence.

In addition to synthetic polymers, six other substances were detected (see Fig. 6C, D). The most common of them were plant material and cellulose, followed by wool, chitin, fur and linen.

Further ANOSIM analysis of the substances other than synthetic polymers demonstrated the significant impact of species on their distribution (ANOSIM statistic R: 0.044, *p* = 0.019). Like in the case of synthetic polymers, the interspecific differences, though statistically significant, were quite subtle. Finally, a multilevel pattern analysis did not reveal any group of species that exhibited a unique distribution pattern for these compounds.

## Discussion

The morphological types (shapes) of microplastics provide crucial information about their dynamics and origins^39^. Overall, the most common morphological type of microplastic detected in the studied amphibian larvae was a fibre. This observation aligns well with findings from tadpole research^17–19^. The proportion of fibres in the overall microplastic composition of tadpoles of *Rana arvalis* (61%) and *Rana temporaria* (57%) that we observed bears a striking resemblance to the 57.1% reported by Karaoğlu and Gül^40^ for the congeneric *Rana macrocnemis.* Additionally, for the *Pelophylax esculentus* complex, the figure was 62% (see Fig. 2A), whereas the aforementioned researchers recorded a figure of 63.6% for the congeneric *Pelophylax ridibundus*. These similarities might be attributed to the shared feeding ecologies among the congeners, in conjunction with similar characteristics of background (water and sediment) microplastic pollution. Interestingly, fibres were found to be the dominant morphological type of microplastics extracted from digestive tracts of *Natrix natrix* and *Natrix tessellata,* representing ca. 94% and 88% of all microplastics, respectively^41^. These snake species, in particular *N. natrix*, are known to feed on both larval and adult frogs^42^, which provides support for the hypothesis that at least some of these microplastic fibres came with prey amphibian items. The shapes of microplastics, along with their colours and chemical compositions, can serve as a pollution fingerprint. Therefore, spatial comparisons (e.g. between compartments of the environment) and diachronic analyses of such fingerprints serve as powerful tools for recognizing dynamics of this emerging class of pollutants. In a previous study of ours, which analyzed MPs extracted not only from amphibian larvae (as in this paper) but also from the water and sediment of the respective water bodies^43^, we identified statistically significant similarities in the shape and colour of the microplastics between those extracted from amphibian larvae and water. Although to a lesser extent, a comparable pattern was observed between MPs extracted from amphibian larvae and sediment.

We found differences in length between different morphological types of microplastics, with a fibre being on average the longest. Hu et al^18^. reported the average length of microplastics to be directly proportional to the developmental stage of tadpoles. In our study however, no impact was observed from factors such as artificial land cover (a proxy of anthropopressure), larval family (taxonomic rank), larval size, or larval MP burden. But, a more detailed examination of our model indicated that larval size had a significant positive relationship with the length of granules and flakes but not with the length of fibres or fragments (see Fig. 3A). This could explain the absence of a significant effect of the larval size on the length of microplastics in general. Our finding might be attributed to larger (and perhaps older) amphibian larvae possessing wider mouths, enabling the ingestion of larger items. Fibre exceptionality could be due to its negligible width and, therefore, the ease of ingestion, regardless of larval size. Furthermore, our findings indicated an inverse relationship between the proportion of artificial land cover within buffers and the lengths of two morphological types of microplastics—flakes and granules, as illustrated in Fig. 3B. However, it is worth noting that microplastics may further breakdown into smaller pieces during their transit through the gastrointestinal tract, a phenomenon documented in crabs^44^.

The surface area of microplastics is relevant because it coinfluences their fate and the toxicological consequences they might lead to^45^. Nevertheless, surface roughness and sorption behaviours also play important roles^46^. Having measured the surface areas of microplastics on the basis of 2D photographs, we observed that larval size had a positive relationship with the surface area of granules and flakes, whereas a negative relationship was evident for fragments (see Fig. 4A). Intuitively, larger larvae are capable of ingesting bigger microplastics, suggesting the presence of a yet unidentified factor causing fragments to deviate from this trend. Additionally, our proxy of anthropopressure was negatively associated with the surface area of both flakes and granules, yet showed a positive association with the surface area of fragments (see Fig. 4B). This pattern is consistent with our observation of an inverse relationship between the share of artificial land cover and the lengths of flakes and granules. Such a trend could imply that under increased anthropopressure, flakes undergo a more pronounced fragmentation, leading to smaller particles. Moreover, a fresh influx of fragments (related to the proximity of pollution sources) could be responsible for the increased average surface area observed for this morphological type of microplastics. Nevertheless, these bold hypotheses need to be tested in future studies. It is also important to note that the intensity of MPs’ fragmentation is influenced not only by their morphological attributes but also by the chemical composition, colour of the particles, temperature fluctuations, sunlight exposure, mechanical stress, and biological factors^47^, which should be considered in future research.

A vast array of microplastic sources leads to environmental microplastics displaying a wide range of colours. The colours are not irrelevant, as these traits can make MPs more attractive to be purposefully ingested by predators^48,49^. Furthermore, colour can offer insights into the origin of microplastics^1^ and present an additional risk to biota due to harmful dyes and pigments^50^. On the other hand, a bias exists in relying on bright colours for visual microplastic identification, which leads to the overestimation of colourful (e.g. pink, blue) particles in comparison to the uncoloured ones^51^. However, colours have seldom been reported in other studies and even less often in relation to the shapes of the MPs^52^. Nevertheless, a review of freshwater microplastic studies that did report on colour showed that transparent (35%), white (21.8%), and blue (16.7%) microplastic colours were the most common ones in water^52^. In sediment samples from freshwater ecosystems, the most frequent MP colour was white, accounting for 38.5%^52^. We found no statistically significant interspecific differences in the colour composition of the bioaccumulated microplastics. Nevertheless, specific colours were overrepresented in certain taxa. In our study, variations in colour composition were evident across different morphological types of microplastics. To the best of our knowledge, this pattern has not yet been reported for microplastics extracted from amphibian larvae. Our finding of blue colour being dominant contrasts with the findings of Karaoğlu and Gül^40^. However, these authors studied a much smaller sample of amphibian larvae. Also, it should be noted that degradation of mesoplastics and macroplastics into microplastics is colour specific. For instance, blue plastic degrades faster due to its ineffective absorbance of ultraviolet radiation^53^. This could partly explain the overrepresentation of blue microplastics that we found. Furthermore, microplastics change colour when exposed to environmental conditions^3^, such as ultraviolet light or other weathering agents; therefore, this phenomenon might obscure the validity of the data and shall be accounted for.

The behaviour of a microplastic particle in water is largely determined by its density, which is in turn influenced by chemical composition. This therefore impacts its fate and the possibility of being ingested. Additionally, the type of polymer that a microplastic is made of can provide insights into its origin. According to the review of López-Rojo et al.^15^, the most frequently detected microplastics in wild tadpoles are nylon fibres. Moreover, polypropylene and polyethylene are known to dominate among the MPs in water, sediment, fish, and macroinvertebrates^54^. Additionally, polyethylene terephthalate and polystyrene microplastics are also commonly detected in both water and sediment^54^. In our study, among the synthetic polymers that we identified, the most common were polyethylene, polypropylene, and poly(ethylene:propylene:diene). While the former two are well-documented pollutants of freshwater ecosystems that have also been previously detected in tadpoles^17,18,40^, the occurrence of poly(ethylene:propylene:diene) was notable. This compound constituted 22% of our synthetic polymer identifications, yet, as far as we are aware, it has not been documented in amphibians. Nonetheless, this synthetic rubber was reported in water^55^, sediment^56^, and significantly, it was found to be prevalent in benthic freshwater invertebrates such as Asellidae, Naididae, and Chironomidae larvae^57^, all of which are known to be preyed upon by amphibian larvae^58^. This suggests the possibility of a trophic transfer of this compound, a hypothesis that warrants further investigation. In addition to synthetic polymers, the substances which we detected included plant material and cellulose. While plant material is undoubtedly natural, cellulose's origin can be dual^59^: it might come from anthropogenic sources such as textiles, or from natural sources such as algae.

## Conclusions and perspectives

Our study contributes to the growing but still limited^60^ body of data on microplastics extracted from wild amphibians, data which shall feed laboratory experiments. Hopefully, a deeper recognition of patterns pertaining to ingestion, elimination of MPs, and consequent health implications could bolster the efficacy of evidence-based amphibian conservation strategies. Besides informing experimental studies, comprehensive characterisation of microplastic particles extracted from amphibians in terms of their chemical composition, size, shape and colouration can provide substantial insights into potential sources of these contaminants, thereby proving valuable to policymaking and identifying priorities in prevention and mitigation measures. Furthermore, by comparing the MP contamination profiles of various environmental matrices, such as water and sediment, with those of species that exhibit a range of feeding habits, we can better understand the potential role of amphibians in the transfer of microplastics. This comparison might also indicate any predilection of amphibian larvae to select certain microplastics under specific circumstances. However, further research into the MP pollution of particular compartments of the environment (invertebrates, algae, water, and sediment) is needed to better understand ecosystem functions (e.g. food webs) under these unprecedented circumstances. Another facet of microplastic ecotoxicology that should not be overlooked is (i) the toxicological effects of leached additives such as plasticisers, the most common of which are phthalate esters (e.g. bisphenol A), known for their ability to disrupt the endocrine system^15^, and (ii) the phenomenon of bioadhesion, an interaction proven in fish but unchartered with respect to amphibians^61^. Lastly, we want to emphasise the importance of studying multiple concurrent stressors.

### Supplementary Information

Supplementary Information.

## Data Availability

The primary datasets generated and analysed during the current study are available from the corresponding author on reasonable request.
