# Supplementary material for: Variation in microplastic characteristics among amphibian larvae: a comparative study across different species and the influence of human activity
Source: Sci Rep. 2024 Jun 12;14:13574. doi: 10.1038/s41598-024-61432-5 (PMC11169503; doi:10.1038/s41598-024-61432-5)
Supplement: Supplementary file 1 — Supplementary Information. [file 41598_2024_61432_MOESM1_ESM.docx]

**Variation in Microplastic Characteristics Among Amphibian Larvae: A Comparative Study Across Different Species and the Influence of Human Activity**

Michał Szkudlarek, Bartłomiej Najbar, Łukasz Jankowiak

Supplementary Table S1. Microplastic counts for each species. This table displays the minimum (Min.), maximum (Max.), and average (Avg.) along with standard deviation (SD) of microplastic counts per species. The average and standard deviation values were rounded to the nearest thousandth. The analyses were based on a total of 934 individuals.

| Species | Min. | Max. | Avg. ± SD |
| --- | --- | --- | --- |
| *Bufo bufo* | 0 | 22 | 4.516 ± 3.566 |
| *Bufotes viridis* | 0 | 13 | 3.267 ± 2.857 |
| *Epidalea calamita* | 0 | 18 | 2.709 ± 2.453 |
| *Rana arvalis* | 0 | 8 | 1.614 ± 1.654 |
| *Pelophylax esculentus* complex | 0 | 5 | 1.444 ± 1.326 |
| *Rana temporaria* | 0 | 17 | 1.586 ± 2.259 |
| *Ichthyosaura alpestris* | 0 | 10 | 0.633 ± 1.224 |
| *Lissotriton vulgaris* | 0 | 11 | 1.075 ± 1.595 |
| *Triturus cristatus* | 0 | 5 | 1.164 ± 1.119 |
| *Bombina bombina* | 0 | 4 | 1.217 ± 1.061 |

Supplementary Table S2. Results of testing model terms regarding the length of microplastics. Significant results are marked in bold.

| Effects | Sum Sq | Mean Sq | NumDF | DenDF | F value | Pr(>F) |
| --- | --- | --- | --- | --- | --- | --- |
| family | 3.559 | 1.186 | 3 | 23.859 | 1.371 | 0.276 |
| size | 0.010 | 0.010 | 1 | 13.273 | 0.011 | 0.918 |
| shape | 2,357.951 | 785.984 | 3 | 1,812.417 | 908.164 | **<0.001** |
| MP burden | 0.790 | 0.790 | 1 | 929.553 | 0.912 | 0.340 |
| Artificial land cover | 0.178 | 0.178 | 1 | 17.521 | 0.206 | 0.656 |
| Dependent variable: log(length) | | | | | | |
| Model structure: size + shape + MP burden + family + Artificial land cover + (1 + size \| family:site) | | | | | | |
| “family” refers to the taxonomic rank of amphibian larvae  MP burden stands for the total number of microplastics extracted from a given individual | | | | | | |

Supplementary Table S3. Predicted values of microplastic length (μm) for their different morphological types (shapes), and post-hoc comparisons (Tukey tests). Significant results are marked in bold.

| Shape | Response | | SE | | df | lower.CL | |  |  |  |
| --- | --- | --- | --- | --- | --- | --- | --- | --- | --- | --- |
| fibre | 839.98 | | 65.46 | | 30.14 | 716.41 | |  |  |  |
| flake | 95.52 | | 8.30 | | 45.29 | 80.19 | |  |  |  |
| fragment | 65.94 | | 5.52 | | 41.33 | 55.68 | |  |  |  |
| granule | 108.58 | | 16.13 | | 336.64 | 81.07 | |  |  |  |
| Contrast | | Ratio | | SE | | | df | | t.ratio | p.value |
| fibre / flake | | 8.794 | | 0.576 | | | 1,810.680 | | 33.208 | **<0.001** |
| fibre / fragment | | 12.738 | | 0.695 | | | 1,793.102 | | 46.648 | **<0.001** |
| fibre / granule | | 7.736 | | 1.081 | | | 1,827.985 | | 14.642 | **<0.001** |
| flake / fragment | | 1.449 | | 0.107 | | | 1,787.120 | | 5.018 | **<0.001** |
| flake / granule | | 0.880 | | 0.128 | | | 1,826.163 | | -0.882 | 0.814 |
| fragment / granule | | 0.607 | | 0.087 | | | 1,828.086 | | -3.490 | **0.003** |

Supplementary Table S4. Results of testing model with interactive terms regarding the length of microplastics. Significant results are marked in bold.

| Effects | Sum Sq | Mean Sq | NumDF | DenDF | F value | Pr(>F) |
| --- | --- | --- | --- | --- | --- | --- |
| MP burden | 1.198 | 1.198 | 1 | 963.589 | 1.425 | 0.233 |
| family | 2.470 | 0.823 | 3 | 22.208 | 0.979 | 0.420 |
| size:shape | 21.465 | 7.155 | 3 | 1,803.605 | 8.507 | **<0.001** |
| shape:Artificial land cover | 21.901 | 7.300 | 3 | 1,804.815 | 8.680 | **<0.001** |
| Dependent variable: log(length)  Model structure: size + shape * size + MP burden + family + shape * Artificial land cover + (1 + size \| family:site)  “family” refers to the taxonomic rank of amphibian larvae  MP burden stands for the total number of microplastics extracted from a given individual | | | | | | |
|  | | | | | | |

Supplementary Table S5. Predicted values of microplastic surface area (μm^2^) for different shapes, and post-hoc comparisons (Tukey tests). The response variable and 95% confidence intervals were back transformed. Significant results are marked in bold.

| Shape | Response | df | lower.CL | upper.CL |  |
| --- | --- | --- | --- | --- | --- |
| flake | 3,025 | 19 | 1,928 | 4,893 |  |
| fragment | 1,140 | 19 | 769 | 1,731 |  |
| granule | 4,033 | 68 | 2,179 | 7,915 |  |
| Contrast | Ratio | SE | df | t.ratio | p.value |
| flake / fragment | 0.337 | 0.045 | 760 | 7.428 | **<0.001** |
| flake / granule | -0.091 | 0.084 | 771 | -1.078 | 0.528 |
| fragment / granule | -0.428 | 0.084 | 769 | -5.098 | **<0.001** |

Supplementary Table S6. Results of testing model terms regarding the surface area of microplastics. Significant results are marked in bold.

| Effects | Sum Sq | Mean Sq | NumDF | DenDF | F value | Pr(>F) |
| --- | --- | --- | --- | --- | --- | --- |
| size | 0.001 | 0.001 | 1 | 71.208 | 0.002 | 0.961 |
| MP burden | 0.006 | 0.006 | 1 | 652.782 | 0.023 | 0.880 |
| family | 3.748 | 1.249 | 3 | 21.981 | 4.470 | **0.014** |
| shape | 18.702 | 9.351 | 2 | 774.116 | 33.459 | **<0.001** |
| Artificial land cover | 0.057 | 0.057 | 1 | 25.690 | 0.204 | 0.057 |
| Dependent variable: area_transformed | | | | | | |
| Model structure: size + MP burden + family + shape + Artificial land cover + (1 + size \| family:site) | | | | | | |
| “family” refers to the taxonomic rank of amphibian larvae  MP burden stands for the total number of microplastics extracted from a given individual | | | | | | |

Supplementary Table S7. Predicted values of microplastic surface area (μm^2^) for different species groups (families), and post-hoc comparisons (Tukey tests). Significant results are marked in bold. The response variable and 95% confidence intervals were back transformed.

| Family | Response | df | lower.CL | upper.CL |  |
| --- | --- | --- | --- | --- | --- |
| Bombinatoridae | 3,985 | 9 | 1,117 | 18,783 |  |
| Ranidae | 2,862 | 38 | 1,711 | 4,983 |  |
| Salamandridae | 1,078 | 17 | 599 | 2,046 |  |
| Bufonidae | 3,811 | 21 | 2,169 | 7,032 |  |
| Contrast | Ratio | SE | df | t.ratio | p.value |
| Bombinatoridae / Ranidae | 0.105 | 0.204 | 10.017 | 0.514 | 0.954 |
| Bombinatoridae / Salamandridae | 0.445 | 0.209 | 9.005 | 2.130 | 0.215 |
| Bombinatoridae / Bufonidae | 0.014 | 0.210 | 11.441 | 0.066 | 1.000 |
| Ranidae / Salamandridae | 0.340 | 0.113 | 12.685 | 3.004 | **0.045** |
| Ranidae / Bufonidae | -0.091 | 0.128 | 25.475 | -0.714 | 0.891 |
| Salamandridae / Bufonidae | -0.431 | 0.145 | 21.700 | -2.974 | **0.033** |

Supplementary Table S8. Results of testing model with interactive terms regarding the surface area of microplastics. Significant results are marked in bold.

| Effects | Sum Sq | Mean Sq | NumDF | DenDF | F value | Pr(>F) |
| --- | --- | --- | --- | --- | --- | --- |
| MP burden | 0.012 | 0.012 | 1 | 650.689 | 0.045 | 0.832 |
| family | 4.318 | 1.439 | 3 | 18.413 | 5.356 | **0.008** |
| size:shape | 3.293 | 1.647 | 2 | 751.866 | 6.128 | **0.002** |
| shape:Artificial land cover | 5.201 | 2.600 | 2 | 760.710 | 9.678 | **<0.001** |
| Dependent variable: area_transformed  Model structure: size + shape * size + MP burden + family + shape * Artificial land cover + (1 + size \| family:site)  “family” refers to the taxonomic rank of amphibian larvae  MP burden stands for the total number of microplastics extracted from a given individual | | | | | | |
|  | | | | | | |
|  | | | | | | |

Supplementary Table S9. Abundance and percentage of particular colours of microplastics extracted from each studied taxon, with the total count (sum) and the average percentage for each colour provided.

|  | **black** | **blue** | **brown** | **clear-white-cream** | **crystalline** | **green** | **grey** | **opaque** | **orange** | **pink** | **red** | **tan** | **transparent** | **white** | **yellow** |
| --- | --- | --- | --- | --- | --- | --- | --- | --- | --- | --- | --- | --- | --- | --- | --- |
| **Bb** | 103 (18.3%) | 198 (35.3%) | 38  (6.8%) | 3  (0.5%) | 43  (7.7%) | 1  (0.2%) | 31 (5.5%) | 29 (5.2%) | 19 (3.4%) | 20 (3.6%) | 21 (3.8%) | 13 (2.3%) | 38 (6.8%) | 2  (0.4%) | 1  (0.2%) |
| **Bv** | 72 (18.4%) | 145  (37%) | 19  (4.9%) | 2  (0.5%) | 19  (4.8%) | 2  (0.5%) | 20 (5.1%) | 22 (5.6%) | 11 (2.8%) | 28 (7.1%) | 22 (5.6%) | 9  (2.3%) | 13 (3.3%) | 5  (1.3%) | 3  (0.8%) |
| **Ec** | 55 (17.3%) | 172 (54.1%) | 6  (1.9%) |  | 5  (1.6%) | 2  (0.6%) | 3  (0.9%) | 14 (4.4%) | 4  (1.3%) | 17 (5.3%) | 13 (4.1%) | 12 (3.8%) | 11 (3.5%) | 3  (0.9%) | 1  (0,3%) |
| **Ra** | 6  (4.2%) | 101 (71.1%) | 2  (1.4%) |  | 2  (1.5%) | 8  (5.6%) | 1  (0.7%) |  | 1  (0.7%) | 5  (3.5%) | 12 (8.5%) |  | 3  (2.1%) | 1  (0.7%) |  |
| **Pec** | 1  (0.8%) | 103 (79.2%) | 2  (1.5%) |  | 3  (2.3%) | 5  (3.9%) |  |  | 5  (3.9%) | 3  (2.3%) | 6  (4.6%) |  | 2  (1.5%) |  |  |
| **Rt** | 31 (15.3%) | 84  (41.4%) | 5  (2.5%) | 1  (0.5%) | 25  (12.2%) | 4  (2%) | 12 (5.8%) | 2  (1%) | 3  (1.5%) | 1  (0.5%) | 10 (4.9%) | 6  (3%) | 1  (0.5%) | 16 (7.9%) | 2  (1%) |
| **Ia** | 7  (12.3%) | 27  (47.3%) | 1  (1.8%) |  | 4  (7%) | 3  (5.3%) | 4  (7%) |  | 2  (3.5%) | 4  (7%) | 2  (3.5%) |  | 2  (3.5%) |  | 1  (1.8%) |
| **Lv** | 1  (1%) | 72  (72%) | 1  (1%) |  | 2  (2%) | 6  (6%) | 3  (3%) |  |  | 8  (8%) | 6  6%) | 1  (1%) |  |  |  |
| **Tc** | 3  (3.7%) | 49  (60.6%) | 1  (1.2%) |  | 1  (1.2%) | 8  (9.9%) | 1  (1.2%) |  | 1  (1.2%) | 5  (6.2%) | 11 (13.6%) |  |  |  | 1  (1.2%) |
| **Bob** |  | 10  (35.8%) |  |  | 4  (14.3%) | 2  (7.1%) |  |  |  | 2  (7.1%) | 4 (14.3%) |  | 6 (21.4%) |  |  |
| **sum** | 279 | 961 | 75 | 6 | 108 | 41 | 75 | 67 | 46 | 93 | 107 | 41 | 76 | 27 | 9 |
| **avg.** | 9.1% | 53.4% | 2.3% | 0.2% | 5.5% | 4.1% | 2.9% | 1.6% | 1.8% | 5.1% | 6.9% | 1.2% | 4.3% | 1.1% | 0.5% |

Bufonidae: Bb – Common Toad *Bufo bufo*, Bv – European Green Toad *Bufotes viridis*, Ec – Natterjack Toad *Epidalea calamita*

Ranidae: Ra – Moor Frog *Rana arvalis*, Pec – Water Frogs *Pelophylax esculentus* complex, Rt – Common Frog *Rana temporaria*

Salamandridae: Ia – Alpine Newt *Ichthyosaura alpestris*; Lv – Smooth Newt *Lissotriton vulgaris*, Tc – Crested Newt *Triturus cristatus*

Bob – European Fire-bellied Toad *Bombina bombina*


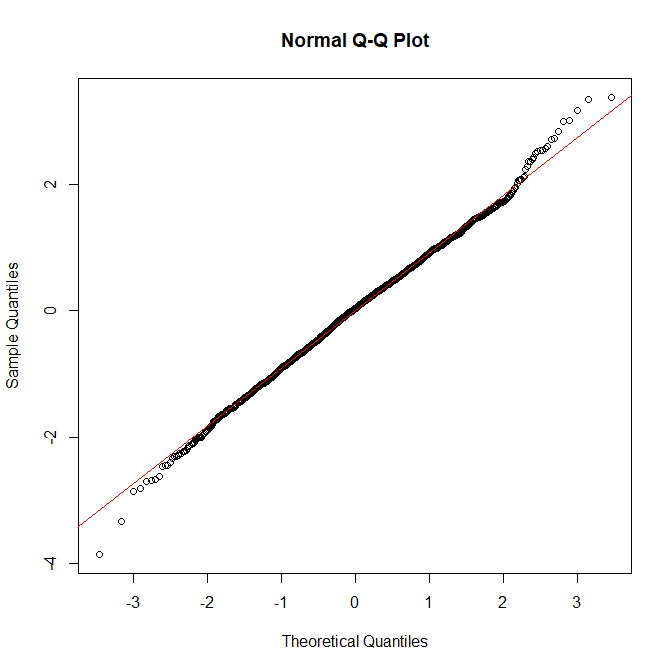


Supplementary Figure S1. Quantile-quantile (Q-Q) plot of residuals from the linear mixed-effects model. It assesses the normality of residuals from the linear mixed-effects model fitted to the log length data.


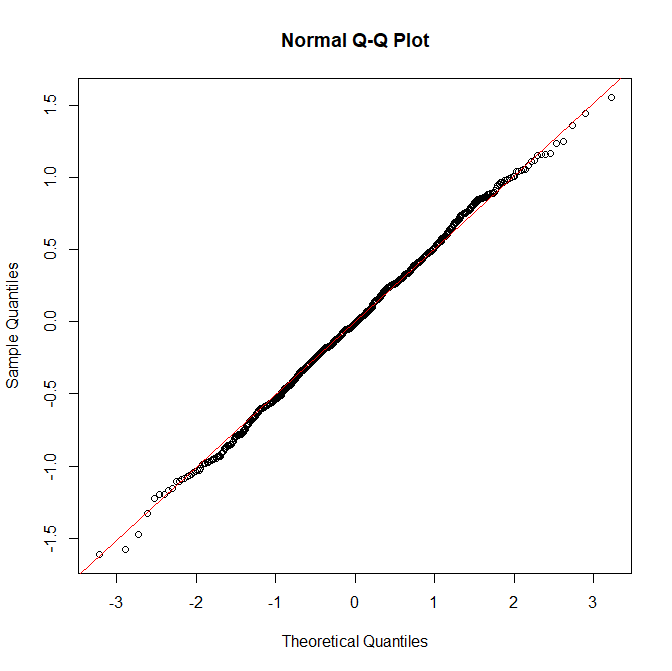


Supplementary Figure S2. Quantile-quantile (Q-Q) plot of residuals from the linear mixed-effects model. It assesses the normality of residuals from the linear mixed-effects model fitted to the Box-Cox transformed area data.

**A**.
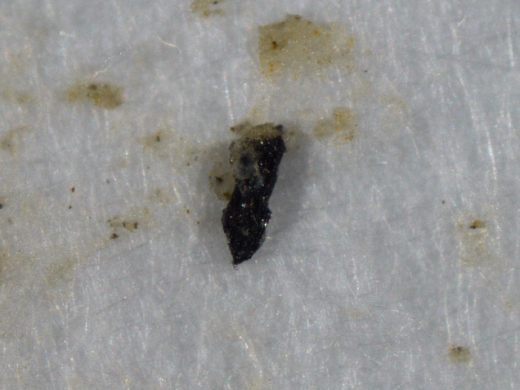
 **B.**
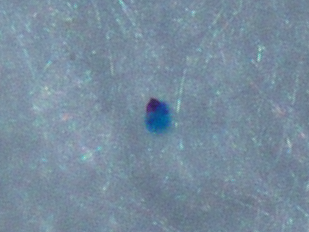


**C.**
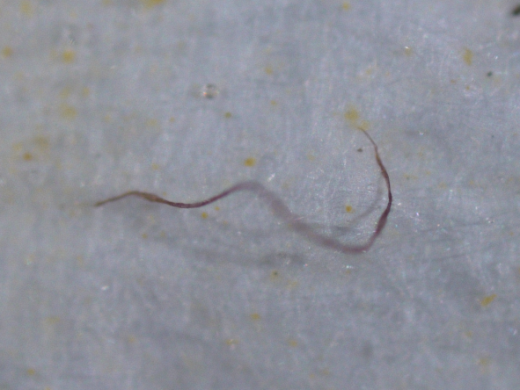
 **D.**
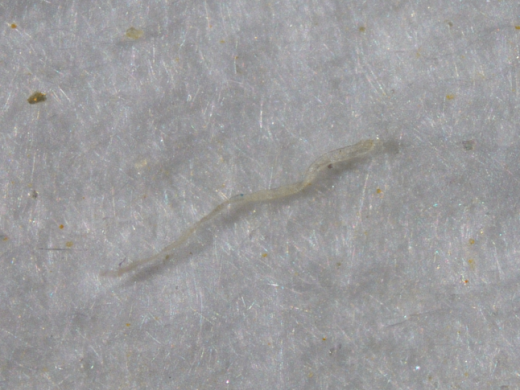


**E.**
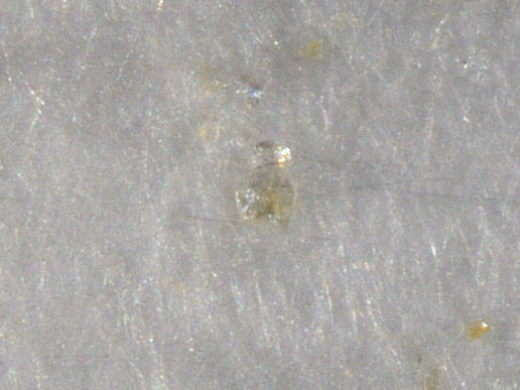
 **F.**
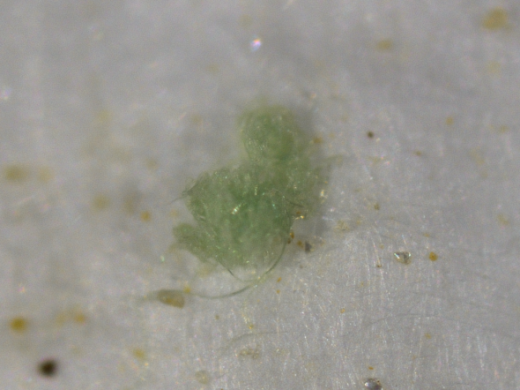


**G.**
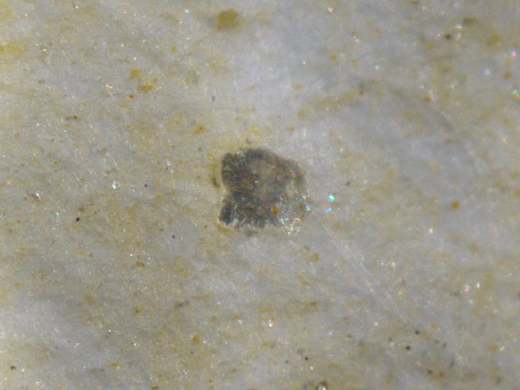
 **H.**
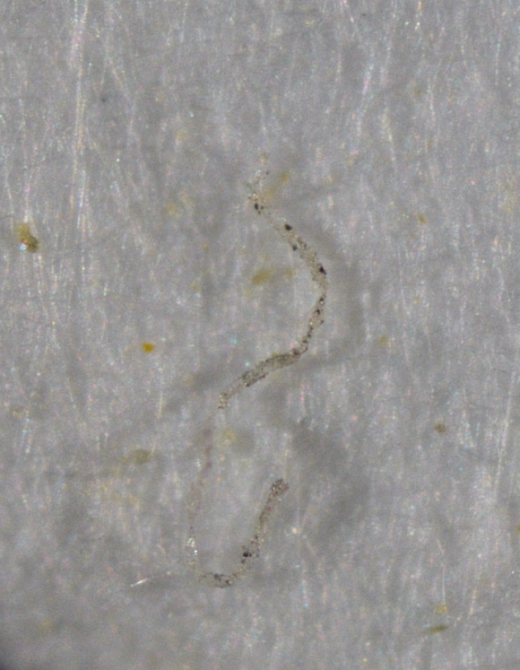


**I.**
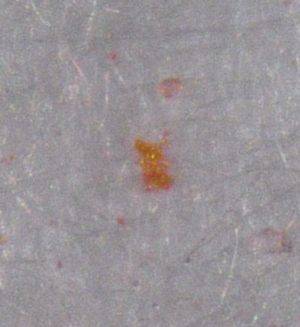
 **J.**
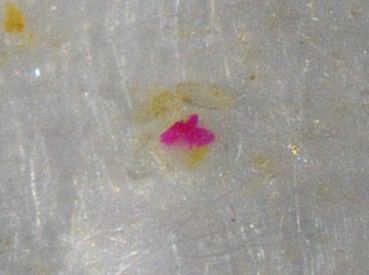


**K.**
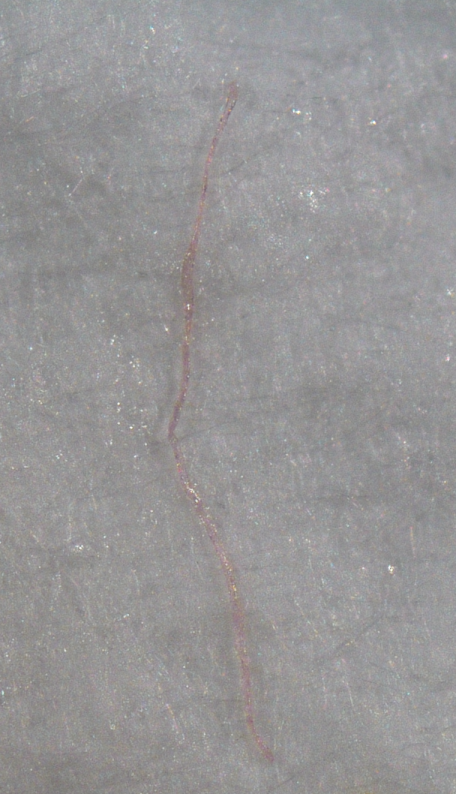
 **L.**
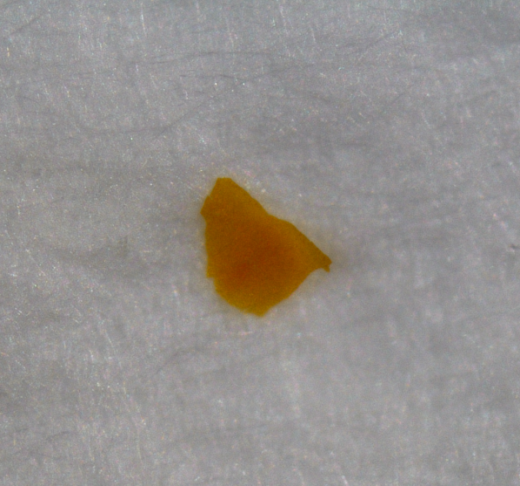


**M.**
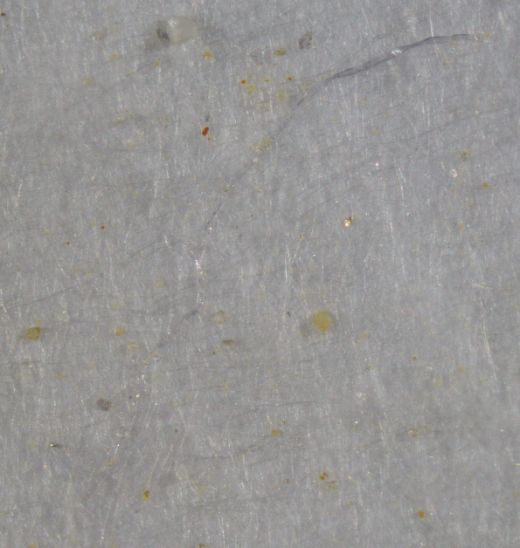
 **N.**
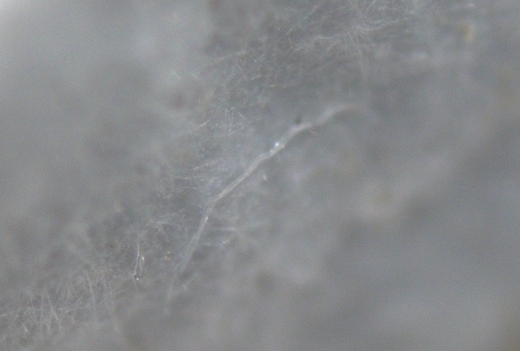


**O.**
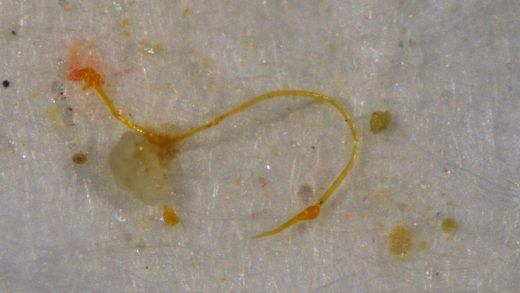


Supplementary Figure S3. Sample microplastics of particular colours: black (**A.**); blue (**B.**); brown (**C.**); clear-white-cream (**D.**); crystalline (**E.**); green (**F.**); grey (**G.**); opaque (**H.**); orange (**I.**); pink (**J.**); red (**K.**); tan (**L.**); transparent (**M.**); white (**N.**); yellow (**O.**).
